# Strong convergence theorems for a class of split feasibility problems and fixed point problem in Hilbert spaces

**DOI:** 10.1186/s13660-018-1881-x

**Published:** 2018-10-23

**Authors:** Jinhua Zhu, Jinfang Tang, Shih-sen Chang

**Affiliations:** 1 Department of Mathematics, Yibin University, Yibin, China; 2 Center for General Education, China Medical University, Taichung, Taiwan

**Keywords:** 26A18, 47H04, 47H05, 47H10, Split feasibility, Maximal monotone operators, Inverse strongly monotone operator, Fixed point problems, Strong convergence theorems

## Abstract

In this paper we consider a class of split feasibility problem by focusing on the solution sets of two important problems in the setting of Hilbert spaces. One of them is the set of zero points of the sum of two monotone operators and the other is the set of fixed points of mappings. By using the modified forward–backward splitting method, we propose a viscosity iterative algorithm. Under suitable conditions, some strong convergence theorems of the sequence generated by the algorithm to a common solution of the problem are proved. At the end of the paper, some applications and the constructed algorithm are also discussed.

## Introduction

Many applications of the split feasibility problem (SFP), which was first introduced by Censor and Elfving [1], have appeared in various fields of science and technology, such as in signal processing, medical image reconstruction and intensity-modulated radiation therapy (for more information, see [2, 3] and the references therein). In fact, Censor and Elfving [1] studied SFP in a finite-dimensional space, by considering the problem of finding a point

1.1 $$ x^{*}\in C \quad \text{such that}\quad Ax^{*}\in Q, $$

where *C* and *Q* are nonempty closed convex subsets of $\Bbb {R}^{n}$, and *A* is an $n\times n$ matrix. They introduced an iterative method for solving SFP.

On the other hand, variational inclusion problems are being used as mathematical programming models to study a large number of optimization problems arising in finance, economics, network, transportation and engineering science. The formal form of a variational inclusion problem is the problem of finding $x^{*}\in H$ such that

1.2 $$ 0\in Bx^{*}, $$

where $B:H \to 2^{H}$ is a set-valued operator. If *B* is a maximal monotone operator, the elements in the solution set of problem (1.2) are called the zeros of this maximal monotone operator. This problem was introduced by Martinet [4], and later it has been studied by many authors. It is well known that the popular iteration method that was used for solving problem (1.2) is the following proximal point algorithm: for a given $x\in H$,

$$ x_{n+1}=J^{B}_{\lambda_{n}}x_{n}, \quad \forall n \in \Bbb {N}, $$

where $\{\lambda_{n}\}\subset (0,\infty)$ and $J^{B}_{\lambda_{n}}=(I+ \lambda_{n}B)^{-1}$ is the resolvent of the considered maximal monotone operator *B* corresponding to $\lambda_{n}$ (see, also [5–9] for more details).

In view of SFP and the fixed point problem, very recently, Montira et al. [10] considered the problem of finding a point $x^{*}\in H$ such

1.3 $$ 0\in Ax^{*}+ Bx^{*} \quad \text{and}\quad Lx^{*} \in F(T), $$

where $A:H_{1}\to H_{1}$ is a monotone operator, and $B:H_{1}\to 2^{H _{1}}$ is a maximal monotone operator, $L:H_{1}\to H_{2}$ is a bounded linear operator and $T:H_{2}\to H_{2}$ is a nonexpansive mapping.

They considered the following iterative algorithm: for any $x_{0} \in H_{1}$,

1.4 $$ x_{n+1}=J^{B}_{\lambda_{n}} \bigl((I-\lambda_{n}A)- \gamma_{n}L^{*}(I-T)L \bigr)x _{n}, \quad \forall n \in \Bbb {N}, $$

where $\{\lambda_{n}\}$ and $\{\gamma_{n}\}$ satisfy some suitable control conditions, and $J^{B}_{\lambda_{n}}$ is the resolvent of a maximal monotone operator *B* associated to $\lambda_{n}$, and proved that sequence (1.4) weakly converges to a point $x^{*}\in \Omega^{A+B} _{L,T}$, where $\Omega^{A+B}_{L,T}$ is the solution set of problem (1.3).

Motivated by the work of Montira et al. [10] and the research in this direction, the purpose of this paper is to study the following split feasibility problem and fixed point problem: find $x^{*} \in H$ such that

1.5 $$ 0\in Ax^{*}+ Bx^{*},\qquad Lx^{*}\in F(T) \quad \text{and}\quad x ^{*} \in F (S), $$

where *A*, *B*, *L* are the same as in (1.3) and $S : H_{1} \to H_{1}$ is a nonexpansive mapping. By using a modified forward–backward splitting method, we propose a viscosity iterative algorithm (see (3.4) below). Under suitable conditions, some strong convergence theorems of the sequence generated by the algorithm to a zero of the sum of two monotone operators and fixed point of mappings are proved. At the end of the paper, some applications and the constructed algorithm are also discussed. The results presented in the paper extend and improve the main results of Montira et al. [10], Byrne et al. [11], Takahashi et al. [12] and Passty [13].

## Preliminaries

Throughout this paper, we denote by $\Bbb {N}$ the set of positive integers, and by $\Bbb {R}$ the set of real numbers. Let *H* be a real Hilbert space with the inner product $\langle \cdot,\cdot \rangle $ and norm $\|\cdot \|$, respectively. When $\{x_{n}\}$ is a sequence in *H*, we denote the weak convergence of $\{x_{n}\}$ to *x* in *H* by $x_{n}\rightharpoonup x$.

Let $T:H\to H$ be a mapping. We say that *T* is a Lipschitz mapping if there exists an $L > 0$ such that

$$ \Vert Tx-Ty \Vert \leq L \Vert x-y \Vert , \quad \forall x,y\in H. $$

The number *L*, associated with *T*, is called a Lipschitz constant. If $L =1$,we say that *T* is a nonexpansive mapping, that is,

$$ \Vert Tx-Ty \Vert \leq \Vert x-y \Vert , \quad \forall x,y\in H. $$

We say that *T* is firmly nonexpansive if

$$ \langle Tx-Ty,x-y\rangle \geq \Vert Tx-Ty \Vert ^{2}, \quad \forall x,y \in H. $$

A mapping $T:H\to H$ is said to be an averaged mapping if it can be written as the average of the identity *I* and a nonexpansive mapping, that is,

2.1 $$ T=(1-\alpha)I+\alpha S, $$

where $\alpha \in (0,1)$ and $S:H\to H$ is a nonexpansive mapping [14]. More precisely, when (2.1) holds, we say that *T* is *α*-averaged. It should be observed that a mapping is firmly nonexpansive if and only if it is a $\frac{1}{2}$-averaged mapping.

Let $A:H\to H$ be a single-valued mapping. For a positive real number *β*, we say that *A* is *β*-inverse strongly monotone (*β*-ism) if

$$ \langle Ax-Ay,x-y\rangle \geq \beta \Vert Ax-Ay \Vert ^{2}, \quad \forall x,y \in H. $$

We now collect some important conclusions and properties, which will be needed in proving our main results.

### Lemma 2.1

([15, 16])

*The following conclusions hold*:

(i) *The composition of finitely many averaged mappings is averaged*. *In particular*, *if* $T_{i}$ *is* $\alpha_{i}$-*averaged*, *where* $\alpha_{i} \in (0,1)$ *for* $i =1,2$, *then the composition* $T_{1}T_{2}$ *is* *α*-*averaged*, *where* $\alpha =\alpha_{1}+\alpha_{2}-\alpha_{1} \alpha_{2}$.
(ii) *If* *A* *is* *β*-*ism and* $\gamma \in (0,\beta ]$, *then* $T:= I-\gamma A$ *is firmly nonexpansive*.
(iii) *A mapping* $T:H\to H$ *is nonexpansive if and only if* $I-T$ *is* $\frac{1}{2}$-*ism*.
(iv) *If* *A* *is* *β*-*ism*, *then*, *for* $\gamma >0$, *γA* *is* $\frac{\beta }{\gamma }$-*ism*.
(v) *T* *is averaged if and only if the complement* $I-T$ *is* *β*-*ism for some* $\beta >\frac{1}{2}$. *Indeed*, *for* $\alpha \in (0,1)$, *T* *is* *α*-*averaged if and only if* $I-T$ *is* $\frac{1}{2 \alpha }$-*ism*.

### Lemma 2.2

([17])

*Let* $T=(1-\alpha)A+\alpha N$ *for some* $\alpha \in (0,1)$. *If* *A* *is* *β*-*averaged and* *N* *is nonexpansive then* *T* *is* $\alpha +(1-\alpha)\beta $-*averaged*.

Let $B:H\to 2^{H}$ be a set-valued mapping. The effective domain of *B* is denoted by $D(B)$, that is, $D(B)=\{x\in H:Bx\neq \emptyset \}$. Recall that *B* is said to be monotone if

$$ \langle x-y,u-v\rangle \geq 0 , \quad \forall x,y\in D(B),u\in Bx,v \in By. $$

A monotone mapping *B* is said to be maximal if its graph is not properly contained in the graph of any other monotone operator. For a maximal monotone operator $B:H\to 2^{H}$ and $r>0$, its resolvent $J^{B}_{r}$ is defined by

$$ J^{B}_{r}:=(I+rB)^{-1}:H\to D(B). $$

It is well known that, if *B* is a maximal monotone operator and *r* is a positive number, then the resolvent $J^{B}_{r}$ is single-valued and firmly nonexpansive, and $F(J^{B}_{r})=B^{-1}0\equiv \{x\in H:0\in Bx \}$, $\forall r>0$ (see [12, 18, 19]).

### Lemma 2.3

([20])

*Let* *H* *be a Hilbert space and let* *B* *be a maximal monotone operator on H*. *Then for all* $s,t >0 $ *and* $x\in H$,

$$\begin{aligned} &\frac{s-t}{s}\langle J_{s}x-J_{t}x,J_{s}x-x \rangle \geq \Vert J_{s}x-J _{t}x \Vert ^{2}; \\ & \Vert J_{s}x-J_{t}x \Vert \leq \bigl( \vert s-t \vert /s \bigr) \Vert x-J_{s}x \Vert . \end{aligned}$$

### Lemma 2.4

([12])

*Let* $H_{1}$ *and* $H_{2}$ *be Hilbert spaces*. *Let* $L:H_{1}\to H_{2}$ *be a nonzero bounded linear operator and* $T:H_{2}\to H_{2}$ *be a nonexpansive mapping*. *If* $B:H_{1}\to 2^{H_{1}}$ *is a maximal monotone operator*, *then*

- $L^{*}(I-T)L$ *is* $\frac{1}{2\|L\|^{2}}$-*ism*,
- *For* $0< r<\frac{1}{\|L\|}$,
- $I-rL^{*}(I-T)L$ *is* $r\|L\|^{2}$-*averaged*,
- $J^{B}_{\lambda }(I-rL^{*}(I-T)L)$ *is* $\frac{1+r\|L\|^{2}}{2}$-*averaged*, *for* $\lambda >0$,
- *If* $r=\|L\|^{-2}$, *then* $I-rL^{*}(I-T)L$ *is nonexpansive*.

### Lemma 2.5

([21])

*Let* $B:H\to 2^{H}$ *be a maximal monotone operator with the resolvent* $J^{B}_{\lambda }=(I+\lambda B)^{-1}$ *for* $\lambda >0$. *Then we have the following resolvent identity*:

$$ J^{B}_{\lambda }x=J^{B}_{\mu } \biggl( \frac{\mu }{\lambda }x+ \biggl(1-\frac{\mu }{ \lambda } \biggr)J^{B}_{\lambda }x \biggr), $$

*for all* $\mu >0$ *and* $x\in H$.

### Lemma 2.6

([22])

*Let* *C* *be a closed convex subset of a Hilbert space* *H* *and let* *T* *be a nonexpansive mapping of* *C* *into itself*. *Then* $U:=I-T$ *is demiclosed*, *i*.*e*., $x_{n}\rightharpoonup x_{0}$ *and* $Ux_{n}\to y_{0}$ *imply* $Ux_{0}=y_{0}$.

### Lemma 2.7

([10])

*Let* $H_{1}$ *and* $H_{2}$ *be Hilbert spaces*. *Let* $A: H_{1}\to H_{1}$ *be a* *β*-*ism*, $B: H_{1}\to 2^{H_{1}}$ *a maximal monotone operator*, $T:H_{2}\to H_{2}$ *a nonexpansive mapping and* $L: H_{1}\to H_{2}$ *a bounded linear operator*. *If* $\Omega^{A+B}_{L,T} \neq \emptyset $, *then the following are equivalent*:

(i) $z\in \Omega^{A+B}_{L,T}$,
(ii) $z=J^{B}_{\lambda }((I_{\lambda }-A)-\gamma L^{*}(I-T)L)z$,
(iii) $0\in L^{*}(I-T)Lz+(A+B)z$,

*where* $\lambda,\gamma >0$ *and* $z\in H_{1}$.

### Lemma 2.8

([23])

*Let* $\{a_{n}\}$ *be a sequence of nonnegative real numbers such that*

$$ a_{n+1}\leq (1-\beta_{n})a_{n}+ \delta_{n}, \quad \forall n\geq 0, $$

*where* $\{\beta_{n}\}$ *is a sequence in* $(0,1)$ *and* $\{\delta_{n}\}$ *is a sequence in* $\Bbb {R}$ *such that*

(i) $\sum^{\infty }_{n=1}\beta_{n}=\infty$;
(ii) $\limsup_{n\to \infty }\frac{\delta_{n}}{\beta_{n}}\leq 0$ *or* $\sum^{\infty }_{n=1}|\delta_{n}|<\infty $.

*Then* $\lim_{n\to \infty }a_{n}=0$.

## Main results

We are now in a position to give the main result of this paper.

### Lemma 3.1

*Let* $H_{1}$ *and* $H_{2}$ *be two real Hilbert spaces*. *Let* $A: H_{1}\to H_{1}$ *be a* *β*-*ism*, $B:H_{1} \to 2^{H_{1}}$ *be a maximal monotone operator*, $T: H_{2}\to H_{2}$ *be a nonexpansive mapping*, *and* $L:H_{1}\to H_{2}$ *be a bounded linear operator*. *Let* $S: H_{1}\to H_{1}$ *be a nonexpansive mapping such that* $F(S)\cap \Omega^{A+B}_{L,T}\neq \emptyset $, *where*

3.1 $$ \Omega^{A+B}_{L,T} : = \bigl\{ x\in (A+B)^{-1} (0) \cap L^{-1} F(T) \bigr\} $$

*is the set of solutions of problem* (1.3). *Let* $f: H_{1}\to H_{1}$ *be a contraction mapping with a contractive constant* $\alpha \in (0,1)$. *For any* $t\in (0,1]$, *let* $W_{t}: H_{1} \to H_{1}$ *be the mapping defined by*

3.2 $$ W_{t}x=tf(x)+(1-t)S \bigl[J^{B}_{\lambda_{n}} \bigl((I- \lambda_{n}A)-\gamma_{n}L ^{*}(I-T)L \bigr)x \bigr], \quad \forall x\in H_{1}, $$

*where* $L^{*}$ *is the adjoint of* *L* *and the sequences* $\lambda_{n}$ *and* $\gamma_{n}$ *satisfy the following control conditions*:

(i) $0< a\leq \lambda_{n}\leq b_{1}<\frac{\beta }{2}$,
(ii) $0< a\leq \gamma_{n}\leq b_{2}<\frac{1}{2\|L\|^{2}}$, *for some* $a,b_{1},b_{2}\in \Bbb {R}$.

*Then* $W_{t}$ *is a contraction mapping with a contractive constant* $[1-t(1-\alpha)]$. *Therefore* $W_{t}$ *has a unique fixed point for each* $t \in (0, 1)$.

### Proof

Note that, for each $n\in \Bbb {N}$, we have

$$ (I-\lambda_{n}A)-\gamma_{n}L^{*}(I-L)L= \frac{1}{2}(I-2\lambda_{n}A)+ \frac{1}{2} \bigl(I-2 \gamma_{n}L^{*}(I-T)L \bigr). $$

Also, by condition (i) and Lemma 2.1(ii), we know that $I-2\lambda _{n}A$ is a firmly nonexpansive mapping, and this implies that $I-2\lambda_{n}A$ must be a nonexpansive mapping. On the other hand, by Lemma 2.4(iia), we know that $I-2\gamma_{n}L^{*}(I-T)L$ is $2\gamma _{n}\|L\|^{2}$-averaged. Thus, by condition (ii) and Lemma 2.2, we see that $(I-\lambda_{n}A)-\gamma_{n}L^{*}(I-T)L$ is $\frac{1+2\gamma_{n} \|L\|^{2}}{2}$-averaged.

Set

3.3 $$ T_{n}:=J^{B}_{\lambda_{n}} \bigl((I-\lambda_{n}A)- \gamma_{n}L^{*}(I-T)L \bigr), \quad \forall n \ge 1. $$

Since $J^{B}_{\lambda_{n}}$ is $\frac{1}{2}$-averaged, by Lemma 2.1(i) we see that $T_{n}$ is $\frac{3+2\gamma_{n}\|L\|^{2}}{4}$-averaged and hence it is nonexpansive. Further, for any $x,y\in H_{1}$, we obtain

$$\begin{aligned} \Vert W_{t}x-W_{t}y \Vert = {}& \bigl\Vert tf(x)+(1-t)ST_{n}x-tf(y)-(1-t)ST_{n}y \bigr\Vert \\ \leq {}&t \bigl\Vert f(x)-f(y) \bigr\Vert +(1-t) \Vert ST _{n}x-ST_{n}y \Vert \\ \leq {}& t\alpha \Vert x-y \Vert +(1-t) \Vert x-y \Vert \\ ={}& \bigl(1-t(1- \alpha) \bigr) \Vert x-y \Vert . \end{aligned}$$

Since $0<1-t(1-\alpha)<1$, it follows that $W_{t}$ is a contraction mapping. Therefore, by Banach contraction principle, $W_{t}$ has a unique fixed point $x_{t}$ in $H_{1}$. □

### Theorem 3.2

*Let* $H_{1}$, $H_{2}$, *A*, *B*, *T*, *L*, *S*, *f* *be the same as in Lemma *3.1. *For any given* $x_{0}\in H_{1}$, *let* $\{u_{n}\}$ *and* $\{x_{n}\}$ *be the sequences generated by*

3.4 $$ \textstyle\begin{cases} u_{n}= J^{B}_{\lambda_{n}} ((I- \lambda_{n}A)-\gamma_{n}L^{*}(I-T)L )x _{n}, \\ x_{n+1}=\alpha_{n}f(x_{n})+(1- \alpha_{n})Su_{n}, \end{cases}\displaystyle \quad \forall n \ge 0, $$

*where* $\{\alpha_{n}\}$ *is a sequence in* $(0,1)$ *such that* $\lim_{n\to \infty }\alpha_{n}=0,\sum^{\infty }_{n=0}\alpha_{n}=\infty $ *and* $\sum^{\infty }_{n=1}|\alpha_{n}-\alpha_{n-1}|<\infty $ *and* $L^{*}$ *is the adjoint of* *L*.

*If* $F(S)\cap \Omega^{A+B}_{L,T}\neq \emptyset $ *and the sequences* $\{\lambda_{n}\}$ *and* $\{\gamma_{n}\}$ *satisfy the following conditions*:

(i) $0< a\leq \lambda_{n}\leq b_{1}<\frac{\beta }{2}$, *and* $\Sigma^{\infty }_{n=1}|\lambda_{n}-\lambda_{n-1}|<\infty$,
(ii) $0< a\leq \gamma_{n}\leq b_{2}<\frac{1}{2\|L\|^{2}}$, *and* $\Sigma^{\infty }_{n=1}|\gamma_{n}-\gamma_{n-1}|<\infty$, *for some* $a, b_{1}, b_{2} \in \Bbb {R}$,

*then the sequences* $\{u_{n}\}$ *and* $\{x_{n}\}$ *both converge strongly to* $z\in F(S)\cap \Omega^{A+B}_{L,T}$, *where* $z=P_{F(S)\cap \Omega^{A+B}_{L,T}}f(z)$, *i*.*e*., *z* *is a solution of problem* (1.5).

### Proof

Take

$$ T_{n}:=J^{B}_{\lambda_{n}} \bigl((I-\lambda_{n}A)- \gamma_{n}L^{*}(I-T)L \bigr), $$

for each $n\in \Bbb {N}$. By Lemma 2.7, we have $\Omega^{A+B}_{L,T}=F(T _{n})$, for all $n\in \Bbb {N}$. Thus, for each $n\in \Bbb {N}$, we can write $x_{n+1}=\alpha_{n}f(x_{n})+(1-\alpha_{n})ST_{n}x_{n}$. By the proof of Lemma 3.1, we see that $T_{n}$ is $\frac{3+2\gamma_{n}\|L\|^{2}}{4}$-averaged. Thus, for each $n\in \Bbb {N}$, we can write

$$ T_{n}=(1- \xi_{n})I+ \xi_{n}V_{n}, $$

where $\xi_{n}=\frac{3+2\gamma_{n}\|L\|^{2}}{4}$ and $V_{n}$ is a nonexpansive mapping. Consequently, we also have $\Omega^{A+B}_{L,T}=F(T _{n})=F(V_{n})$, for all $n\in \Bbb {N}$. Using this fact, for each $p\in F(S)\cap \Omega^{A+B}_{L,T}$, we see that

3.5 $$\begin{aligned} \Vert u_{n}-p \Vert ^{2}= {}& \Vert T_{n} x_{n}-p \Vert ^{2} \\ ={}& \bigl\Vert (1-\xi_{n})x_{n}+\xi_{n}V _{n}x_{n}-p \bigr\Vert ^{2} \\ ={}& \bigl\Vert (1-\xi_{n}) (x_{n}-p)+ \xi_{n}(V_{n} x_{n}-p) \bigr\Vert ^{2} \\ ={}&(1-\xi_{n}) \Vert x_{n}-p \Vert ^{2}+ \xi_{n} \Vert V_{n}x_{n}-p \Vert ^{2}- \xi_{n}(1- \xi_{n}) \Vert x_{n}-V_{n}x_{n} \Vert ^{2} \\ \leq {}& \Vert x_{n}-p \Vert ^{2}-\xi_{n}(1- \xi _{n}) \Vert x_{n}-V_{n}x_{n} \Vert ^{2} \end{aligned}$$

for each $n\in \Bbb {N}$. Since $I-T_{n}=\xi_{n}(I-V_{n})$, in view of (3.5) we get

3.6 $$ \Vert u_{n}-p \Vert ^{2}\leq \Vert x_{n}-p \Vert ^{2}-(1-\xi_{n}) \Vert x_{n}-T_{n}x_{n} \Vert ^{2}, $$

for each $n\in \Bbb {N}$. Since $\xi_{n}=\frac{3+2\gamma_{n}\|L\|^{2}}{4} \in (\frac{3}{4},1)$, we obtain

3.7 $$ \Vert u_{n}-p \Vert ^{2}\leq \Vert x_{n}-p \Vert ^{2}. $$

Next, we estimate

$$\begin{aligned} \Vert x_{n+1}-p \Vert = {}& \bigl\Vert \alpha_{n}f(x_{n})+(1- \alpha_{n})Su_{n}-p \bigr\Vert \\ \leq {}& \alpha_{n} \bigl\Vert f(x_{n})-p \bigr\Vert +(1- \alpha_{n}) \Vert Su_{n}-p \Vert \\ \leq {}& \alpha _{n} \bigl( \bigl\Vert f(x_{n})-f(p) \bigr\Vert + \bigl\Vert f(p)-p \bigr\Vert \bigr)+(1-\alpha_{n}) \Vert u_{n}-p \Vert \\ \leq {}& \alpha_{n}\alpha \Vert x_{n}-p \Vert + \alpha_{n} \bigl\Vert f(p)-p \bigr\Vert +(1-\alpha_{n}) \Vert x _{n}-p \Vert \\ \leq {}& \bigl(1-\alpha_{n}(1-\alpha) \bigr) \Vert x_{n}-p \Vert +\alpha_{n} \bigl\Vert f(p)-p \bigr\Vert \\ \leq {}&\max \biggl\{ \Vert x_{n}-p \Vert ,\frac{ \Vert f(p)-p \Vert }{1-\alpha } \biggr\} . \end{aligned}$$

By induction, we can prove that

3.8 $$ \Vert x_{n+1}-p \Vert \leq \max \biggl\{ \Vert x_{0}-p \Vert ,\frac{ \Vert f(p)-p \Vert }{1-\alpha } \biggr\} , \quad \forall n \ge 0. $$

Hence $\{x_{n}\}$ is bounded and so are $\{u_{n}\}$, $\{f(x_{n})\}$ and $\{Su_{n}\}$.

Next, we show that

3.9 $$ \lim_{n \to \infty } \Vert x_{n+1}-x_{n} \Vert = 0. $$

In fact, it follows from (3.4) that

3.10 $$\begin{aligned} \Vert x_{n+1}-x_{n} \Vert = {}& \bigl\Vert \alpha_{n}f(x_{n})+(1-\alpha_{n})Su_{n}- \bigl(\alpha _{n-1}f(x_{n-1})+(1-\alpha_{n-1})Su_{n-1} \bigr) \bigr\Vert \\ ={}& \bigl\Vert \alpha_{n}f(x_{n})- \alpha_{n}f(x_{n-1})+ \alpha_{n}f(x_{n-1})-\alpha_{n-1}f(x_{n-1})+(1- \alpha_{n})Su_{n} \\ &{}-(1-\alpha_{n})Su_{n-1}+(1-\alpha_{n})Su_{n-1}-(1- \alpha_{n-1})Su_{n-1} \bigr\Vert \\ \leq {}& \alpha_{n}\alpha \Vert x_{n}-x_{n-1} \Vert +(1- \alpha_{n}) \Vert Su_{n}-Su_{n-1} \Vert +2 \vert \alpha_{n}-\alpha_{n-1} \vert K \\ \leq {}& \alpha_{n}\alpha \Vert x_{n}-x_{n-1} \Vert +(1-\alpha_{n}) \Vert u_{n}-u_{n-1} \Vert +2 \vert \alpha_{n}-\alpha_{n-1} \vert K , \end{aligned}$$

where $K:=\sup \{\|f(x_{n})\|+\|Su_{n}\|:n\in \Bbb {N}\}$.

Put

$$\begin{aligned} &y_{n} = \bigl((I-\lambda_{n}A)- \gamma_{n}L^{*}(I-T)L \bigr)x_{n} \quad \text{and} \\ &u_{n} =T_{n}x_{n}=J^{B}_{\lambda_{n}}y_{n}. \end{aligned}$$

Since $J^{B}_{\lambda_{n}}((I-\lambda_{n}A)-\gamma_{n}L^{*}(I-T)L)$ is nonexpansive, it follows from Lemma 2.3 that

3.11 $$\begin{aligned} \Vert u_{n+1}-u_{n} \Vert ={}& \bigl\Vert J^{B}_{\lambda_{n+1}}y_{n+1}-J^{B}_{\lambda _{n}}y_{n} \bigr\Vert \\ \leq{}& \bigl\Vert J^{B}_{\lambda_{n+1}}y_{n+1}-J^{B}_{\lambda _{n+1}} \bigl((I-\lambda_{n+1}A)-\gamma_{n+1}L^{*}(I-T)L \bigr)x_{n} \bigr\Vert \\ &{} + \bigl\Vert J ^{B}_{\lambda_{n+1}} \bigl((I- \lambda_{n+1}A)- \gamma_{n+1}L^{*}(I-T)L \bigr)x_{n}-J ^{B}_{\lambda_{n}}y_{n} \bigr\Vert \\ \leq{}& \Vert x_{n+1}-x_{n} \Vert + \bigl\Vert J^{B}_{\lambda _{n+1}} \bigl((I-\lambda_{n+1}A)- \gamma_{n+1}L^{*}(I-T)L \bigr)x_{n}-J^{B}_{ \lambda_{n}}y_{n} \bigr\Vert \\ \leq {}& \bigl\Vert J^{B}_{\lambda_{n+1}} \bigl((I- \lambda_{n+1}A)- \gamma_{n+1}L^{*}(I-T)L \bigr)x_{n} \\ &{} -J^{B}_{\lambda_{n+1}} \bigl((I- \lambda_{n}A)- \gamma_{n}L^{*}(I-T)L \bigr)x_{n} \bigr\Vert \\ &{} +\big\| J^{B}_{ \lambda_{n+1}}y_{n}-J^{B}_{\lambda_{n}}y_{n}\big\| + \Vert x_{n+1}-x_{n} \Vert \\ \leq{}& \bigl\Vert \bigl((I-\lambda_{n+1}A)-\gamma_{n+1}L^{*}(I-T)L \bigr)x_{n}- \bigl((I-\lambda _{n}A)-\gamma_{n}L^{*}(I-T)L \bigr)x_{n} \bigr\Vert \\ &{} + \bigl\Vert J^{B}_{\lambda_{n+1}}y _{n}-J^{B}_{\lambda_{n}}y_{n} \bigr\Vert + \Vert x_{n+1}-x_{n} \Vert \\ \leq{}& \vert \lambda _{n+1}-\lambda_{n} \vert \Vert Ax_{n} \Vert + \vert \gamma_{n+1}-\gamma_{n} \vert \bigl\Vert L^{*}(I-T)Lx _{n} \bigr\Vert \\ &{} +\frac{ \vert \lambda_{n+1}-\lambda_{n} \vert }{a} \bigl\Vert J^{B}_{ \lambda_{n+1}}y_{n}-y_{n} \bigr\Vert + \Vert x_{n+1}-x_{n} \Vert \\ \leq{}& \Vert x_{n+1}-x _{n} \Vert +M_{1} \vert \lambda_{n+1}-\lambda_{n} \vert +M_{2} \vert \gamma_{n+1}-\gamma_{n} \vert , \end{aligned}$$

where $M_{1}$ and $M_{2}$ are constants defined by

$$\begin{aligned} & M_{1} = \sup_{n} \biggl( \Vert Ax_{n} \Vert +\frac{1}{a} \bigl\Vert J^{B}_{\lambda_{n+1}}y_{n}-y _{n} \bigr\Vert \biggr), \\ & M_{2} = \sup_{n} \bigl\Vert L^{*}(I-T)Lx_{n} \bigr\Vert . \end{aligned}$$

Therefore it follows from (3.10) and (3.11) that

$$ \Vert x_{n+1}-x_{n} \Vert \leq \bigl(1- \alpha_{n}(1-\alpha) \bigr) \Vert x_{n}-x_{n-1} \Vert + M _{1} \vert \lambda_{n+1}-\lambda_{n} \vert +M_{2} \vert \gamma_{n+1}-\gamma_{n} \vert +2 \vert \alpha_{n}-\alpha_{n-1} \vert K. $$

Take

$$\begin{aligned} & \beta_{n}:=\alpha_{n}(1-\alpha)\quad \text{and} \\ &\delta_{n}:=M_{1} \vert \lambda_{n+1}- \lambda_{n} \vert + M_{2} \vert \gamma_{n+1}- \gamma_{n} \vert +2 \vert \alpha _{n}- \alpha_{n-1} \vert K. \end{aligned}$$

It follows from Lemma 2.8 that

3.12 $$ \lim_{n\to \infty } \Vert x_{n+1}-x_{n} \Vert =0. $$

Now, we write

$$\begin{aligned} x_{n+1}-x_{n}= {}&\alpha_{n}f(x_{n})+(1- \alpha_{n})Su_{n}-x_{n} \\ ={}& \alpha_{n} \bigl(f(x_{n})-x_{n} \bigr)+(1- \alpha_{n}) (Su_{n}-x_{n}). \end{aligned}$$

Since $\|x_{n+1}-x_{n}\|\to 0$ and $\alpha_{n}\to 0$ as $n\to \infty $, we obtain

3.13 $$ \lim_{n\to \infty } \Vert Su_{n}-x_{n} \Vert =0. $$

Next, we prove that

$$ \lim_{n \to \infty } \Vert x_{n}-u_{n} \Vert = \lim_{n \to \infty } \Vert x_{n}-T _{n} x_{n} \Vert = 0. $$

In fact, it follows from (3.4) and (3.6) That

$$\begin{aligned} \Vert x_{n+1}-p \Vert ^{2} & = \bigl\Vert \alpha_{n}f(x_{n})+(1-\alpha_{n})Su_{n}-p \bigr\Vert ^{2} \\ &\leq \alpha_{n} \bigl\Vert f(x_{n})-p \bigr\Vert ^{2}+(1-\alpha_{n}) \Vert Su_{n}-p \Vert ^{2} \\ &\leq \alpha_{n} \bigl\Vert f(x_{n})-p \bigr\Vert ^{2}+(1-\alpha_{n}) \Vert u_{n}-p \Vert ^{2} \\ &\leq \alpha_{n} \bigl\Vert f(x_{n})-p \bigr\Vert ^{2}+(1-\alpha_{n}) \bigl( \Vert x_{n}-p \Vert ^{2}-(1- \xi_{n}) \Vert x_{n}-T_{n}x_{n} \Vert ^{2} \bigr) \\ &\leq \alpha_{n} \bigl\Vert f(x_{n})-p \bigr\Vert ^{2}+ \Vert x _{n}-p \Vert ^{2}-(1- \xi_{n}) \Vert x_{n}-T_{n}x_{n} \Vert ^{2}. \end{aligned}$$

Hence, we obtain

$$\begin{aligned} (1-\xi_{n}) \Vert x_{n}-T_{n}x_{n} \Vert ^{2}\leq{} & \alpha_{n} \bigl\Vert f(x_{n})-p \bigr\Vert ^{2}+ \bigl( \Vert x _{n}-p \Vert ^{2}- \Vert x_{n+1}-p \Vert ^{2} \bigr) \\ \leq {}& \alpha_{n} \bigl\Vert f(x_{n})-p \bigr\Vert ^{2}+ \bigl( \Vert x _{n}-p \Vert + \Vert x_{n+1}-p \Vert \bigr) \Vert x_{n}-x_{n+1} \Vert . \end{aligned}$$

Since $\alpha_{n}\to 0$ as $n\to \infty $, and $\xi_{n}=\frac{3+2 \gamma_{n}\|L\|^{2}}{4}\in (\frac{3}{4},1)$, from (3.12) we obtain

3.14 $$ \lim_{n\to \infty } \Vert u_{n}-x_{n} \Vert = \Vert x_{n}-T_{n}x_{n} \Vert =0. $$

Therefore we have

3.15 $$ \Vert Su_{n}-u_{n} \Vert \leq \Vert Su_{n}-x_{n} \Vert + \Vert x_{n}-u_{n} \Vert \to 0, \quad \text{as } n\to \infty. $$

On the other hand, since $\{x_{n}\}$ is bounded, let $\{x_{n_{j}}\}$ be any subsequence of $\{x_{n}\}$ with $x_{n_{j}}\rightharpoonup \hat{x}$. Also, we assume that $\lambda_{n_{j}}\rightarrow \hat{\lambda }\in (0,\frac{ \beta }{2})$ and $\gamma_{n_{j}}\to \hat{\gamma }\in (0, \frac{1}{2\|L\|^{2}})$.

Letting

$$ \hat{T}=J^{B}_{\hat{\lambda }} \bigl((I-\hat{\lambda }A)-\hat{\gamma }L^{*}(I-T)L \bigr), $$

we know that *T̂* is $\frac{3+2\hat{\gamma }\|L\|^{2}}{4}$-averaged and $F(\hat{T})=\Omega^{A+B}_{L,T}$.

Hence, for each $j\in \Bbb {N}$ we have

3.16 $$\begin{aligned} \Vert x_{n_{j}}-\hat{T}x_{n_{j}} \Vert &{}\leq \Vert x_{n_{j}}-u_{n_{j}} \Vert + \Vert T_{n _{j}}x_{n_{j}}- \hat{T}x_{n_{j}} \Vert \\ &{}\leq \Vert x_{n_{j}}-u_{n_{j}} \Vert + \bigl\Vert J ^{B}_{\lambda_{n_{j}}}z_{j}-J^{B}_{\hat{\lambda }}z_{j} \bigr\Vert + \bigl\Vert J^{B}_{ \hat{\lambda }}z_{j}- \hat{T}x_{n_{j}} \bigr\Vert , \end{aligned}$$

where $z_{j}=((I-\lambda_{n_{j}}A)-\gamma_{n_{j}}L^{*}(I-T)L)x_{n_{j}}$. Now, we estimate the last term in (3.16). We have

$$\begin{aligned} \bigl\Vert J^{B}_{\hat{\lambda }}z_{j}- \hat{T}x_{n_{j}} \bigr\Vert ={} & \bigl\Vert J^{B}_{ \hat{\lambda }} \bigl((I-\lambda_{n_{j}}A)-\gamma_{n_{j}}L^{*}(I-T)L \bigr)x_{n _{j}} -J^{B}_{\hat{\lambda }} \bigl((I-\hat{\lambda }A)- \hat{\gamma }L^{*}(I-T)L \bigr)x _{n_{j}} \bigr\Vert \\ \leq{} & \bigl\Vert \bigl((I-\lambda_{n_{j}}A)-\gamma_{n_{j}}L^{*}(I-T)L \bigr)x _{n_{j}} - \bigl((I-\hat{\lambda }A)-\hat{\gamma }L^{*}(I-T)L \bigr)x_{n_{j}} \bigr\Vert \\ \leq{} & \bigl\Vert (\lambda_{n_{j}}-\hat{\lambda })Ax_{n_{j}} \bigr\Vert + \bigl\Vert (\gamma_{n_{j}}- \hat{\gamma })L^{*}(I-T)Lx_{n_{j}} \bigr\Vert \\ \leq{} & \vert \lambda_{n_{j}}- \hat{\lambda } \vert \Vert Ax_{n_{j}} \Vert +2 \vert \gamma_{n_{j}}-\hat{\gamma } \vert \bigl\Vert L^{*} \bigr\Vert \Vert L \Vert \Vert x _{n_{j}}-p \Vert \end{aligned}$$

for each $j\in \Bbb {N}$. This implies that

3.17 $$ \lim_{j\to \infty } \bigl\Vert J^{B}_{\hat{\lambda }}z_{j}- \hat{T}x_{n_{j}} \bigr\Vert =0. $$

Next, we estimate the second term in (3.16). By Lemma 2.5, we have

3.18 $$\begin{aligned} \bigl\Vert J^{B}_{\lambda_{n_{j}}}z_{j}-J^{B}_{\hat{\lambda }}z_{j} \bigr\Vert = {}& \biggl\Vert J^{B} _{\hat{\lambda }} \biggl( \frac{\hat{\lambda }}{\lambda_{n_{j}}}z_{j} + \biggl(1-\frac{ \hat{\lambda }}{\lambda_{n_{j}}} \biggr)J^{B}_{\lambda_{n_{j}}}z_{j} \biggr)-J^{B} _{\hat{\lambda }}z_{j} \biggr\Vert \\ \leq {}& \biggl\Vert \frac{\hat{\lambda }}{\lambda_{n _{j}}}z_{j}+ \biggl(1- \frac{\hat{\lambda }}{\lambda_{n_{j}}} \biggr)J^{B}_{ \lambda_{n_{j}}}z_{j}-z_{j} \biggr\Vert \\ ={}& \biggl\Vert \biggl(1-\frac{\hat{\lambda }}{ \lambda_{n_{j}}} \biggr)J^{B}_{\lambda_{n_{j}}}z_{j}- \biggl(1-\frac{\hat{\lambda }}{ \lambda_{n_{j}}} \biggr)z_{j} \biggr\Vert \\ ={}& \biggl\Vert \biggl(1-\frac{\hat{\lambda }}{\lambda_{n _{j}}} \biggr) \bigl(J^{B}_{\lambda_{n_{j}}}z_{j}-z_{j} \bigr) \biggr\Vert \\ ={}& \biggl\vert 1-\frac{ \hat{\lambda }}{\lambda_{n_{j}}} \biggr\vert \bigl\Vert J^{B}_{\lambda_{n_{j}}}z_{j}-z _{j} \bigr\Vert , \quad \forall j \ge 1. \end{aligned}$$

Also for each $j\in \Bbb {N}$ we have

$$\begin{aligned} \bigl\Vert J^{B}_{\lambda_{n_{j}}}z_{j}-z_{j} \bigr\Vert = {}& \Vert T_{n_{j}}x_{n_{j}}-z_{j} \Vert \\ ={}& \bigl\Vert u_{n_{j}}-x_{n_{j}}+\lambda_{n_{j}}Ax_{n_{j}}+ \gamma_{n_{j}}L ^{*}(I-T)Lx_{n_{j}} \bigr\Vert \\ \leq {}& \Vert u_{n_{j}}-x_{n_{j}} \Vert + \lambda_{n_{j}} \Vert Ax _{n_{j}} \Vert +\gamma_{n_{j}} \bigl\Vert L^{*}(I-T)Lx_{n_{j}} \bigr\Vert \\ \leq {}& \Vert u_{n_{j}}-x _{n_{j}} \Vert + \lambda_{n_{j}} \Vert Ax_{n_{j}} \Vert +2\gamma_{n_{j}} \bigl\Vert L^{*} \bigr\Vert \Vert L \Vert \Vert x _{n_{j}}-p \Vert . \end{aligned}$$

This shows that $\{\|(J^{B}_{\lambda_{n_{j}}}z_{j}-z_{j})\|\}$ is a bounded sequence. This, together with (3.18), implies

3.19 $$ \lim_{j\to \infty } \bigl\Vert J^{B}_{\lambda_{n_{j}}}z_{j}-J^{B}_{ \hat{\lambda }}z_{j} \bigr\Vert =0. $$

Substituting (3.14), (3.17) and (3.19) into (3.16), we get

3.20 $$ \lim_{j\to \infty } \Vert x_{n_{j}}-\hat{T}x_{n_{j}} \Vert =0. $$

Thus, by Lemma 2.6, it follows that $\hat{x}\in F(\hat{T})=\Omega^{A+B} _{L,T}$.

Furthermore, it follows from (3.13) and (3.14) that $\{u_{n}\}$, $\{x_{n}\}$ and $\{S(u_{n})\}$ have the same asymptotical behavior, so $\{u_{n}\}$ also converges weakly to *x̂*. Since *S* is nonexpansive, by (3.13) and Lemma 2.6, we obtain that $\hat{x}\in F(S)$. Thus $\hat{x}\in \Omega^{A+B}_{L,T}\cap F(S)$.

Next, we claim that

3.21 $$ \lim \sup_{n\to \infty } \bigl\langle f(z)-z,x_{n}-z \bigr\rangle \leq 0, $$

where $z=P_{F(S)\cap \Omega^{A+B}_{L,T}}f(z)$.

Indeed, we have

3.22 $$\begin{aligned} \limsup_{n\to \infty } \bigl\langle f(z) -z, x_{n}-z \bigr\rangle ={} & \limsup_{n\to \infty } \bigl\langle f(z)-z,Su_{n}-z \bigr\rangle \\ \leq{} & \limsup_{n\to \infty } \bigl\langle f(z)-z,u_{n}-z \bigr\rangle \\ ={}& \bigl\langle f(z)-z, \hat{x}-z \bigr\rangle \\ \leq {}&0, \end{aligned}$$

since $z=P_{F(S)\cap \Omega^{A+B}_{L,T}}f(z)$.

Finally, we show that $x_{n}\to z$. Indeed, we have

$$\begin{aligned} \Vert x_{n+1}-z \Vert ^{2}= {}& \bigl\langle \alpha_{n}f(x_{n})+(1-\alpha_{n})Su_{n}-z,x _{n+1}-z \bigr\rangle \\ ={}&\alpha_{n} \bigl\langle f(x_{n})-z,x_{n+1}-z \bigr\rangle +(1- \alpha_{n})\langle Su_{n}-z,x_{n+1}-z \rangle \\ \leq {}&\alpha_{n} \bigl\langle f(x_{n})-z,x_{n+1}-z \bigr\rangle +(1-\alpha_{n})\langle u_{n}-z,x _{n+1}-z\rangle \\ \leq{} &\alpha_{n} \bigl\langle f(x_{n})-f(z),x_{n+1}-z \bigr\rangle +\alpha_{n} \bigl\langle f(z)-z,x_{n+1}-z \bigr\rangle \\ &{}+(1-\alpha_{n}) \langle x_{n}-z,x_{n+1}-z\rangle \\ \leq{} &\frac{\alpha_{n}}{2} \bigl\{ \bigl\Vert f(x)-f(z) \bigr\Vert ^{2}+ \Vert x _{n+1}-z \Vert ^{2} \bigr\} + \alpha_{n} \bigl\langle f(z)-z,x_{n+1}-z \bigr\rangle \\ &{}+\frac{(1- \alpha_{n})}{2} \bigl\{ \Vert x_{n}-z \Vert ^{2}+ \Vert x_{n+1}-z \Vert ^{2} \bigr\} \\ \leq {}& \frac{1}{2} \bigl(1-\alpha_{n} \bigl(1- \alpha^{2} \bigr) \bigr) \Vert x_{n}-z \Vert ^{2}+\frac{(1-\alpha _{n})}{2} \Vert x_{n+1}-z \Vert ^{2} \\ &{}+\frac{\alpha_{n}}{2} \Vert x_{n+1}-z \Vert ^{2}+ \alpha_{n} \bigl\langle f(z)-z,x_{n+1}-z \bigr\rangle , \end{aligned}$$

which implies that

$$ \Vert x_{n+1}-z \Vert ^{2}\leq \bigl(1- \alpha_{n} \bigl(1-\alpha^{2} \bigr) \bigr) \Vert x_{n}-z \Vert ^{2}+2 \alpha_{n} \bigl\langle f(z)-z,x_{n+1}-z \bigr\rangle . $$

Now, by using (3.22) and Lemma 2.8, we deduce that $x_{n}\to z$. Further it follows from $\|u_{n}-x_{n}\|\to 0,u_{n}\rightharpoonup \hat{x} \in F(S)\cap \Omega^{A+B}_{L,T}$ and $x_{n}\to z$ as $n\to \infty $, that $z=\hat{x}$. This completes the proof. □

If $A:=0$, the zero operator, then the following result can be obtained from Theorem 3.2 immediately.

### Corollary 3.3

*Let* $H_{1}$ *and* $H_{2}$ *be Hilbert spaces*. *Let* $B : H_{1} \to 2^{H_{1}}$ *be a maximal monotone operator*, $T : H_{2} \to H_{2}$ *a nonexpansive mapping and* $L : H_{1}\to H_{2}$ *a bounded linear operator*. *Let* $S: H_{1}\to H_{1}$ *be a nonexpansive mapping such that* $\Gamma =F(S)\cap B^{-1}(0)\cap L^{-1}(F(T))\neq \emptyset $. *Let* $f: H_{1}\to H_{1}$ *be a contraction mapping with a contractive constant* $\alpha \in (0,1)$. *For any given* $x_{0}\in H_{1}$, *let* $\{u_{n}\}$ *and* $\{x_{n}\}$ *be the sequences generated by*

3.23 $$ \textstyle\begin{cases} u_{n}= J^{B}_{\lambda_{n}}( (I- \gamma_{n}L^{*}(I-T)L )x_{n}, \\ x_{n+1}= \alpha_{n}f(x_{n})+(1- \alpha_{n})Su_{n}, \end{cases}\displaystyle \forall n \ge 0. $$

*If the sequences* $\{\alpha_{n}\}$, $\{\lambda_{n}\}$ *and* $\{\gamma _{n}\}$ *satisfy all the conditions in Theorem *3.2, *then the sequences* $\{u_{n}\}$ *and* $\{x_{n}\}$ *both converge strongly to* $z=P_{\Gamma }f(z)$ *which is a solution of problem* (1.5) *with* $A =0$.

If $H_{1}=H_{2}$, $L=I$, then by applying Theorem 3.2, we can obtain the following result.

### Corollary 3.4

*Let* $H_{1}$ *be Hilbert spaces*. *Let* $A : H_{1} \to H_{1}$ *be a* *β*-*ism and* $B : H_{1} \to 2^{H_{1}}$ *be a maximal monotone operator*. *Let* $S: H_{1}\to H_{1}$ *be a nonexpansive mapping such that* $\Gamma_{1}=F(S)\cap (A+B)^{-1}0\cap F(T)\neq \emptyset $. *Let* $f: H_{1}\to H_{1}$ *be a contraction mapping with constant* $\alpha \in (0,1)$. *For any* $x_{0}\in H_{1}$ *arbitrarily*, *let the iterative sequences* $\{u_{n}\}$ *and* $\{x_{n}\}$ *be generated by*

3.24 $$ x_{n+1}=\alpha_{n}f(x_{n})+(1-\alpha_{n})SJ^{B}_{\lambda_{n}} \bigl((I- \lambda_{n}A)-\gamma_{n}(I-T) \bigr)x_{n}. $$

*If the sequences* $\{\alpha_{n}\}$, $\{\lambda_{n}\}$ *and* $\{\gamma _{n}\}$ *satisfy all the conditions in Theorem *3.2, *then the sequences* $\{u_{n}\}$ *and* $\{x_{n}\}$ *both converge strongly to* $z\in \Gamma _{1}$, *where* $z=P_{\Gamma_{1}} f(z)$.

## Applications

In this section, we will utilize the results presented in the paper to study variational inequality problems, convex minimization problem and split common fixed point problem in Hilbert spaces.

### Application to variational inequality problem

Let *C* be a nonempty closed and convex subset of a Hilbert space *H*. Recall that the normal cone to *C* at $u\in C$ is defined by

$$ N_{C}(u)= \bigl\{ z\in H:\langle z,y-u\rangle \leq 0, \forall y\in C \bigr\} . $$

It is well known that $N_{C}$ is a maximal monotone operator. In the case $B:=N_{C}:H\to 2^{H}$ we can verify that the problem of finding $x^{*}\in H$ such that $0\in Ax^{*}+Bx^{*}$ is reduced to the problem of finding $x^{*}\in C$ such that

4.1 $$ \bigl\langle Ax^{*},x-x^{*} \bigr\rangle \geq 0, \quad \forall x\in C. $$

In the sequel, we denote by $\operatorname{VIP}(C,A)$ the solution set of problem (4.1). In this case, we also have $J^{B}_{\lambda }= P_{C}$ (the metric projection of *H* onto *C*). By the above consideration, problem (1.5) is reduced to finding

4.2 $$ x^{*}\in \operatorname{VIP}(C,A) \quad \text{such that }Lx^{*}\in F(T) \text{ and } x ^{*} \in F(S). $$

Therefore, the following convergence theorem can be immediately obtained from Theorem 3.2.

#### Theorem 4.1

*Let* $H_{1}$ *and* $H_{2}$ *be Hilbert spaces*. *Let* $A : H_{1} \to H_{1}$ *be a* *β*-*ism operator*, $T : H_{2}\to H_{2}$ *a nonexpansive mapping and* $L : H_{1}\to H_{2}$ *a bounded linear operator*. *Let* $S: H_{1}\to H_{1}$ *be a nonexpansive mapping such that* $F(S)\cap \Omega^{A,C}_{L,T}\neq \emptyset $, *where*

$$ \Omega^{A,C}_{L,T}: = \operatorname{VIP}(C,A) \cap L^{-1} \bigl(F(T) \bigr). $$

*Let* $f: H_{1}\to H_{1}$ *be a contraction mapping with a contractive constant* $\alpha \in (0,1)$. *For any given* $x_{0}\in H_{1}$, *let the sequences* $\{u_{n}\}$ *and* $\{x_{n}\}$ *be generated by*

4.3 $$ \textstyle\begin{cases} u_{n}= P_{C} ((I-\lambda_{n}A)- \gamma_{n}L^{*}(I-T)L )x_{n}, \\ x_{n+1}= \alpha_{n}f(x_{n})+(1- \alpha_{n})Su_{n}, \end{cases} $$

*where* $\{\alpha_{n}\}$ *is a sequence in* $(0,1)$ *such that* $\lim_{n\to \infty }\alpha_{n}=0$, $\sum^{\infty }_{n=0}\alpha_{n}=\infty$, $\sum^{\infty }_{n=1}|\alpha_{n}-\alpha_{n-1}|<\infty$, $L^{*}$ *is the adjoint of* *L*, *and the sequences* $\{\lambda_{n}\}$ *and* $\{\gamma_{n}\}$ *satisfy conditions* (*i*)*–*(*ii*) *in Theorem *3.2. *Then the sequences* $\{u_{n}\}$ *and* $\{x_{n}\}$ *both converge strongly to* $z=P_{F(S)\cap \Omega^{A,C}_{L,T}}f(z)$, *which is a solution of problem* (4.2).

### Application to convex minimization problem

Let $g:H\to R$ be a convex function, which is also Fréchet differentiable. Let *C* be a given closed convex subset of *H*. In this case, by setting $A:=\nabla g$, the gradient of *g*, and $B = N_{C}$, the problem of finding $x^{*}\in (A+B)^{-1}0$ is equivalent to finding a point $x^{*}\in C$ such that

4.4 $$ \bigl\langle \nabla g \bigl(x^{*} \bigr), x-x^{*} \bigr\rangle \geq 0, \quad \forall x \in C. $$

Note that (4.4) is equivalent to the following minimization problem: find $x^{*}\in C$ such that

$$ x^{*}\in \arg \min_{x\in C}g(x). $$

Thus, in this situation, problem (1.5) is reduced to the problem of finding

4.5 $$ x^{*}\in \arg \min_{x\in C}g(x)\quad \text{such that }Lx^{*}\in F(T) \text{ and } x^{*} \in F(S). $$

Denote by

$$ \Omega^{g,C}_{L,T}: = \arg \min_{x\in C}g(x) \cap L^{-1} \bigl(F(T) \bigr). $$

Then, by using Theorem 3.2, we can obtain the following result.

#### Theorem 4.2

*Let* $H_{1}$ *and* $H_{2}$ *be Hilbert spaces and let* *C* *be a nonempty closed convex subset of* $H_{1}$. *Let* $g: H_{1} \to \Bbb {R}$ *be a convex and Fréchet differentiable function*, ∇*g* *be* *β*-*Lipschitz*, $T:H_{2}\to H_{2}$ *be a nonexpansive mapping*, *and let* $L : H_{1}\to H_{2}$ *be a bounded linear operator*. *Let* $S: H_{1}\to H_{1}$ *be a nonexpansive mapping such that* $F(S)\cap \Omega^{g,C}_{L,T}\neq \emptyset $. *Let* $f: H_{1}\to H_{1}$ *be a contraction mapping with a contractive constant* $\alpha \in (0,1)$. *For any given* $x_{0}\in H_{1}$, *let* $\{u_{n}\}$ *and* $\{x_{n}\}$ *be the sequences generated by*

4.6 $$ \textstyle\begin{cases} u_{n}= P_{C} ((I-\lambda_{n} \nabla g)-\gamma_{n}L^{*}(I-T)L )x_{n}, \\ x _{n+1}=\alpha_{n}f(x_{n})+(1- \alpha_{n})Su_{n}, \end{cases}\displaystyle \forall n \ge 0. $$

*If the sequences* $\{\alpha_{n}\}$, $\{\lambda_{n}\}$ *and* $\{\gamma _{n}\}$ *satisfy all the conditions in Theorem *3.2, *then the sequences* $\{u_{n}\}$ *and* $\{x_{n}\}$ *both converge strongly to* $z\in F(S) \cap \Omega^{g,C}_{L,T}$, *where* $z=P_{F(S)\cap \Omega^{g,C}_{L,T}}f(z)$, *which is a solution of problem* (4.5).

#### Proof

Note that if $g: H\to \Bbb {R}$ is convex and $\nabla g:H \to H$ is *β*-Lipschitz continuous for $\beta >0$ then ∇*g* is $\frac{1}{\beta }$-ism (see [24]). Thus, the required result can be obtained immediately from Theorem 3.2. □

### Application to split common fixed point problem

Let $V:H_{1}\to H_{1}$ be a nonexpansive mapping. Then, by Lemma 2.1(iii), we know that $A:=I-V$ is $\frac{1}{2}$-ism. Furthermore, $Ax^{*}=0$ if and only if $x^{*}\in F(V)$. Hence problem (1.5) can be reduced to the problem of finding

4.7 $$ x^{*}\in F(V) \quad \text{such that } Lx^{*}\in F(T) \text{ and } x^{*} \in F(S), $$

where $T:H_{2}\to H_{2}$, $L:H_{1}\to H_{2}$ and $S: H_{1} \to H_{1}$ are mappings as in Theorem 3.2.

This problem is called the split common fixed point problem (SCFP), and was studied by many authors (see [25–28], for example). By using Theorem 3.2, we can obtain the following result.

#### Theorem 4.3

*Let* $H_{1}$ *and* $H_{2}$ *be Hilbert spaces*. *Let* $V:H_{1} \to H_{1}$ *and* $T : H_{2}\to H_{2}$ *be nonexpansive mappings and* $L : H_{1}\to H_{2}$ *a bounded linear operator*. *Let* $S: H_{1} \to H_{1}$ *be a nonexpansive mapping such that* $F(S)\cap \Omega ^{V}_{L,T}\neq \emptyset$, *where*

$$ \Omega^{V}_{L,T}: = F(V) \cap L^{-1} \bigl(F(S) \bigr). $$

*Let* $f: H_{1}\to H_{1}$ *be a contraction mapping with a contractive constant* $\alpha \in (0,1)$. *For any given* $x_{0}\in H_{1}$, *let be* $\{u_{n}\}$ *and* $\{x_{n}\}$ *be the iterative sequences generated by*

4.8 $$ \textstyle\begin{cases} u_{n}= (I-\lambda_{n})x_{n}+ \lambda_{n}Vx_{n}-\gamma_{n}L^{*}(I-T)Lx _{n}, \\ x_{n+1}=\alpha_{n}f(x_{n})+(1- \alpha_{n})Su_{n}, \end{cases}\displaystyle \forall n \ge 0, $$

*where the sequences* $\{\alpha_{n}\}$, $\{\lambda_{n}\}$ *and* $\{\gamma_{n}\}$ *satisfy all the conditions in Theorem *3.2. *Then the sequences* $\{u_{n}\}$ *and* $\{x_{n}\}$ *both converge strongly to a point* $z=P_{F(S)\cap \Omega^{V}_{L,T}}f(z)$, *which is a solution of problem* (4.7).

#### Proof

We consider $B:=0$, the zero operator. The required result follows from the fact that the zero operator is monotone and continuous, hence it is maximal monotone. Moreover, in this case, we see that $J^{B}_{\lambda }$ is the identity operator on $H_{1}$, for each $\lambda >0$. Thus algorithm (3.4) reduces to (4.8), by setting $A:=I-V$ and $B:=0$. □
